# Zebrafish in oncology

**DOI:** 10.18632/aging.100746

**Published:** 2013-05-16

**Authors:** Jorge Barriuso, Raghavendar Nagaraju, Adam Hurlstone

**Affiliations:** Faculty of life Sciences, The University of Manchester, Manchester M13 9PT, UK

Novel high-throughput technologies generate an unprecedented amount of data, which present a challenge for oncologists aiming to provide personalized treatment. Progress in genetic engineering and xenotransplantation in conjunction with live imaging could establish the zebrafish as one of the most versatile animal models for cancer research. In a recent review article, we discussed the current and potential future role of zebrafish models in cancer research (1). In addition to the advantages of the zebrafish in the field of translational oncology which are the main focus of our previously published review, the zebrafish is flourishing as a tool to tackle complex cancer related processes, such as tumour heterogeneity, the link between aging and cancer and the relationship between inflammation and the tumour.

Regarding heterogeneity, a technique entailing co-injection of two distinct melanoma cell lines into the pericardial space of zebrafish embryos combined with live imaging revealed how different clones with different capabilities cooperate to invade and spread through the zebrafish embryo (2). Furthermore, a transgenic strain named *Zebrabow* was developed to visualise heterogeneity within the zebrafish, comprising three genetically-encoded fluorescent reporter proteins (RFP, CFP and YFP) that can be spectrally resolved flanked by two distinct and mutually exclusive *loxp* sequences. The results published by Pan et al showed how Cre recombinase-mediated recombination lead to combinatorial expression of the fluorescent proteins in neighbouring cells, allowing different lineages of cells to be traced during development (3). This technology could readily be utilised to study heterogeneity within zebrafish transgenic cancer models.

Zebrafish telomeres have an average length of 10-20 kb, similar to human telomeres. It is known that telomerase activity is involved not only in aging but in cancer. The activity of telomerase in cancer cells is not only induced to avoid the shortening of telomeres but also protects mitochondrial DNA from damage (4). As shown by Anchelin and colleagues, telomerase-deficient fish showed a p53-dependant premature aging and reduced lifespan in the first generation, as occurs in humans but not in mice (5). This study also showed how a fish model for premature aging could be further used to study the interactions between ageing and cancer.

Further data supporting the zebrafish as optimal for investigating the regulation of telomerase activity were provided by Sun et al. They identified the zebrafish orthologue of the Human LPTS/PinX1, a negative regulator of telomerase activity, and confirmed its role as a telomerase inhibitor in human cell lines and in zebrafish (6).

Zebrafish provide unrivalled opportunity to evaluate the interaction between cancer cells and their microenvironment. Recently, a creative example of engrafting human cells in zebrafish embryos was used to address the relationship between macrophages and human breast cancer cells. The study demonstrated the feasibility to use freshly isolated Tumour Associated Macrophages (TAMs) from human samples and co-inject them with different established human cell lines. The authors then used this model to dissect the contribution of TAMs to metastasis. Furthermore, they proposed that the model could give information about the metastatic potential of tumours from where the TAMs were collected (7). This particular study highlights the versatility of the zebrafish in translational oncology research.

The zebrafish community is characterised by the speed with which it adopts new molecular biology techniques developed for other animal models and cell lines and rapidly adapt them to the fish. To illustrate this principle, a flurry of papers implementing CRISPR/Cas9 technology to edit the zebrafish genome has appeared since our review was published. As an example, the Zon lab in Boston have developed a vector carrying both the guide RNA and Cas9 that is able to generate a tissue specific knock down of a gene of interest (8).

In summary, that the zebrafish is a versatile model for cancer research is reinforced even when used to study complex processes associated with cancer behaviour. In our recent review, we mainly focused on the applicability to the translational oncology field of the fish model from a closer-to-the-patient bed perspective. Here, we underlined, as already known by developmental biologists, the zebrafish model's amenability to shed light on a whole rainbow of different cancer-related processes.
